# Combination of preoperative tumour markers and lymphovascular invasion with TNM staging as a cost and labour efficient subtyping of colorectal cancer

**DOI:** 10.1038/s41598-020-66652-z

**Published:** 2020-06-24

**Authors:** Tomoki Yamano, Shinichi Yamauchi, Masataka Igeta, Yuya Takenaka, Jihyung Song, Kei Kimura, Michiko Yasuhara, Akihito Babaya, Kozo Kataoka, Naohito Beppu, Masataka Ikeda, Naohiro Tomita, Kenichi Sugihara

**Affiliations:** 1 Division of Lower GI Surgery, Department of Surgery, Hyogo College of Medicine, Hyogo, Japan; 2 Division of Colorectal Surgery, Department of Gastrointestinal Surgery, Tokyo Medical and Dental University, Tokyo, Japan; 3 Department of Biostatistics, Hyogo College of Medicine, Hyogo, Japan

**Keywords:** Translational research, Surgical oncology, Cancer therapy, Colorectal cancer, Predictive markers, Prognostic markers

## Abstract

Tumour-Node-Metastasis (TNM) staging of colorectal cancer (CRC) needs further classification for better treatment because of disease heterogeneity. Although molecular classifications which are expensive and laborious are under study, cost and labour efficient subtyping is desirable. We assessed the combinations of preoperative tumour marker (TM) elevation and tumour lymphovascular invasion (LVI) as a solution. We used the pooled data of 7151 colon cancer (CC) patients and 4620 rectal cancer (RC) patients who received curative surgery between 2004 and 2008 in Japan. The best-matched subtyping for predicting relapse-free survival (RFS) was statistically selected using the c-index and Akaike’s information criterion. This subtyping (TM-LVI), which consisted of three categories by TM elevation status and severity of LVI status, was an independent prognostic factor for RFS of CC (stage IIa, IIIb, and IIIc) and RC (stage I, IIa, IIb, IIIa, and IIIb) and also for disease specific survival of CC (stage IIa, IIb, IIIb, and IIIc) and RC (all stage except for IIc). Although TM-LVI classified CRC patients into low and high recurrence risk groups, the application of adjuvant therapy was not accordance with the TM-LVI status. TM-LVI may be a cost and labour efficient subtyping of colorectal cancer for better treatment strategy.

## Introduction

The treatments of colorectal cancer (CRC) patients after curative surgery are based on clinical staging. CRC patients with high risk stages (i.e., stages II and III) are recommended for adjuvant therapy^1–5^. However, the heterogeneous characteristics of CRC results in different prognoses among CRC patients within the same clinical stage. As a result, both the American Joint Committee on Cancer/Union for International Cancer Control staging system (Tumour-Node-Metastasis [TNM] staging system) and the Japanese Society for Cancer of the Colon and Rectum (JSCCR) staging system have developed several editions to improve the accuracy of predicting prognosis^6–8^. These staging systems are composed of tumour depth, nodal status, and metastatic status, although there are differences among the versions even in the same staging system. Therefore, further subtyping in each clinical stage is indispensable for better treatment strategy.

Recently, classifications of CRC using genetic background have been proposed as alternative or additional tools for staging. Microsatellite instability (MSI) status indicated good prognosis in patients with stage II/III right-sided colon cancer (CC)^9^. Consensus molecular subtypes consisting of four groups (MSI immune, canonical, metabolic, and mesenchymal) have been presented as powerful tools for CRC biology and treatment^10–13^. These classifications based on genetic analysis have been expected to replace TNM staging in the future, although benefit for treatment decision has not been validated.

However, high risk patients for recurrence have been selected using clinicopathological features. Many articles have demonstrated that elevations in tumour markers (TMs, e.g., carcinoembryonic antigen [CEA] or cancer antigen 19-9 [CA19-9]) have been associated with poor prognosis in CRC^14–18^. Lymphovascular invasion (LVI) was also indicated to be a prognostic factor for CRC^19–22^. However, these factors have not been included in the TNM staging systems and have not been assessed in combination. We considered that improvement of classification by combination of these features was necessary before applying novel classifications that require more labour and cost.

Thus, we statistically selected the most suitable subtyping combined the influence of TM elevation and LVI on relapse-free survival (RFS) using the pooled data collected by the Japanese Study Group for Postoperative Follow-up of CRC (JFUP-CRC), which is one of the largest data collections in Japan^23,24^. We evaluated this classification (so called TM-LVI) as a prognostic factor for RFS and disease specific survival (DSS) in each TNM staging. We also assessed the association between application of adjuvant therapy and TM-LVI status.

## Results

### Clinicopathological characteristics of the patients

Clinicopathological characteristics were compared between CC and rectal cancer (RC) patients. There were significant differences between CC and RC patients in age (*P* < 0.0001), sex (*P* < 0.0001), histological type (*P* < 0.0001), the ratio of CEA elevation (*P* = 0.008), the degree of LVI (*P* < 0.0001), dissected lymph node number (12 ≤ or not), TNM stage (*P* < 0.0001), and the application of adjuvant therapy (*P* < 0.0001) but not in the ratio of CA19-9 elevation (Supplementary Table 1).

Adjuvant therapy consisted of chemotherapy except for two cases of radiotherapy and seven cases of chemoradiotherapy in RC. Most patients (94.6%) received 5-fluorouracil based chemotherapy. A total of 1.6% and 1.1% of the patients received oxaliplatin-based and irinotecan-based chemotherapy, respectively (Supplementary Table 1).

Thus, further analysis was performed by CC and RC.

### Selection of the most suitable subtyping

Among six candidate subtypes (Table 1), ABC1 was the most statistically suitable subtype with the lowest Akaike’s information criterion (AIC, Supplementary Table 2) and the highest Harrell’s concordance index (c-index, Supplementary Table 3) according to the models including TNM staging. Then, we called ABC1 as TM-LVI. Both TNM and TM-LVI were significant in the Cox model for RFS in CC and RC patients. The interaction term between TNM and TM-LVI was significant in RC but not CC. Thus, in ranking the incidence rate of RFS, the main effect model with TNM and TM-LVI was applied to CC, and the interaction model was employed for RC. When RFS was ranked from 1^st^ to 21^st^ by TNM staging and TM-LVI, RFS was not ordered by TNM staging. Stage IIIa was a low recurrence risk group compared to most of stage II (Supplementary Table 4). Category C by TM-LVI belonged to the highest recurrence risk group in each TNM stage.

### Validation of TM-LVI for RFS and DSS

**Figure 1:**
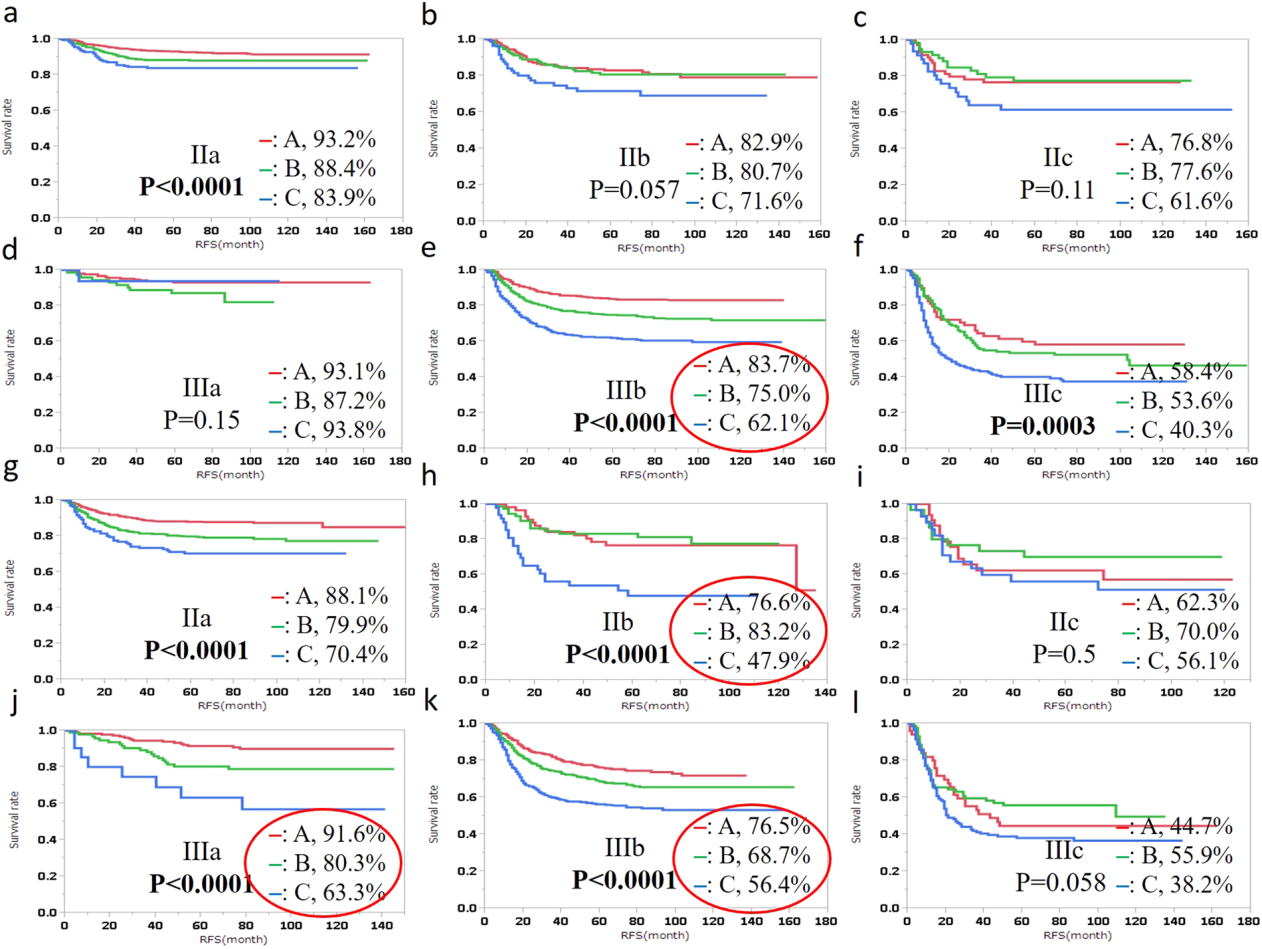
Relapse-free survival (RFS) of colon cancer (**a**–**f**) and rectal cancer patients (**g**-**l**) by TM-LVI status is shown for each TNM stage. The 5-year RFS rate is described on the right of the TM-LVI status. Bold type, P** <** 0.05; Red circle, difference in the RFS rate among TM-LVI statuses > 20%.

**Figure 2:**
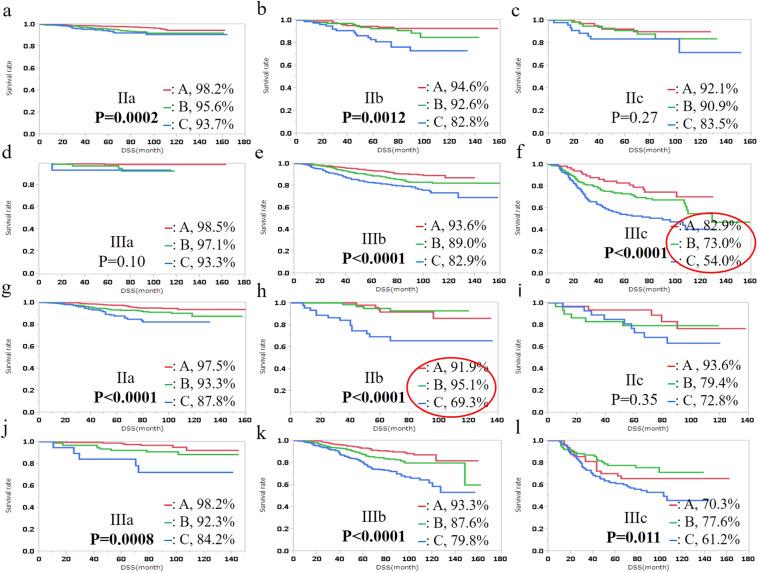
Disease free survival (DSS) of colon cancer (**a**–**f**) and rectal cancer (**g**–**l**) patients by TM-LVI status is shown for each TNM stage. The 5-year DSS rate is described on the right of the TM-LVI status. Bold type, P < 0.05; Red circle, difference of DSS rate among TM-LVI statuses > 20%.

Log-rank test demonstrated that RFS was significantly different by TM-LVI status in CC (stage IIa, IIIb, and IIIc) and RC (stage I, IIa, IIb, IIIa, IIIb) (Fig. 1). In particular, the 5-year RFS differed more than 20% by TM-LVI status between A and C (83.7% and 62.1% in stage IIIb of CC, 76.6% and 47.9% in stage IIb of RC, 91.6% and 63.3% in stage IIIa of RC, and 76.5% and 56.4% in stage IIIb of RC). Log-rank test also demonstrated that DSS was significantly different by TM-LVI in CC (stage IIa, IIb, IIIb, and IIIc) and RC (except for IIc) (Fig. 2). The 5-year DSS differed more than 20% by TM-LVI status between A and C (82.9% and 54.3% in stage IIIc of CC and 91.9% and 69.3% in stage IIb of RC).

We assessed the factors associated with RFS and DSS by univariate and multivariate analysis. TM-LVI was an independent prognostic factor for RFS of CC (stage IIa, IIIb, and IIIc, Tables 1,2) and RC (stage I, IIa, IIb, IIIa, and IIIb, Table 3) and also for DSS of CC (stage IIa, IIb, IIIb, and IIIc, Table 4) and RC (all stage except for IIc, Table 5).

**Table 1:** Candidate subtypes were determined depending on the tumour marker elevation status and lymphovascular invasion status.

Subtypes	Tumour marker elevation	Lymphovascular invasion			
		None	Slight	Mild	Severe
ABC1	Both CEA and CA19-9	B	C	C	C
	Either CEA or CA19-9	A	B	C	C
	None	A	A	B	C
ABC2	Both CEA and CA19-9	B	B	C	C
	Either CEA or CA19-9	A	B	C	C
	None	A	A	B	C
ABC3	Both CEA and CA19-9	A	B	C	C
	Either CEA or CA19-9	A	B	C	C
	None	A	B	B	C
ABC4	Both CEA and CA19-9	A	B	C	C
	Either CEA or CA19-9	A	B	C	C
	None	A	A	B	C
ABC5	Both CEA and CA19-9	B	B	C	C
	Either CEA or CA19-9	B	B	C	C
	None	A	B	B	C
AB	Both CEA and CA19-9	A	A	B	B
	Either CEA or CA19-9	A	A	B	B
	None	A	A	A	B

CA19-9, Cancer antigen 19-9; CEA, Carcinoembryonic antigen.

**Table 2:** Univariate and multivariate analysis for relapse-free survival of colon cancer was performed in each clinical stage.

Stage	I		IIa				IIb		IIc		IIIa		IIIb				IIIc			
Factors	Univariate analysis		Univariate analysis		Multivariate analysis		Univariate analysis		Univariate analysis		Univariate analysis		Univariate analysis		Multivariate analysis		Univariate analysis		Multivariate analysis	
	RR	*P*	RR	*P*	RR	*P*	RR	*P*	RR	*P*	RR	*P*	RR	*P*	RR	*P*	RR	*P*	RR	*P*
Age																				
75≥ /<75	0.97.	0.94	1.22	0.21	—	—	1.3	0.29	1.63	0.10	1.02	0.97	1.12	0.30	—	—	1.12	0.50		
Gender																				
M/F	1.53	0.20	1.25	0.14	—	—	0.96	0.84	1.04	0.88	0.93	0.86	1.29	**0.01**	1.29	**0.01**	1.21	0.17	1.21	0.16
Histology																				
Diff/Undiff	0.94	0.95	6.26	**0.0003**	6.11	**0.0004**	1.51	0.34	0.88	0.78	0.61	0.53	1.11	0.60	—	—	0.95	0.76		
Dissected LN																				
12 > /12≤	0.89	0.71	1.54	**0.01**	1.42	0.11	1.37	0.27	2.13	**0.03**	0.99	0.97	1.41	**0.008**	1.36	0.06	1.30	0.30		
Adjuvant																				
Yes/No	6.25	**0.002**	1.01	0.98	—	—	1.39	0.19	0.80	0.53	0.82	0.62	0.94	0.55	—	—	0.92	0.57		
TM-LVI																				
C/A	1.9×10^−9^	0.42	2.31	**<0.0001**	2.32	**0.0001**	1.80	0.08	1.79	0.14	0.98	0.19	2.84	**<0.0001**	2.84	**<0.001**	1.91	**0.0004**	1.94	**0.0004**
B/A	0.96		1.60		1.54		1.03		0.91		2.17		1.70		1.69		1.19		1.21	
C/B	2.1×10^-9^		1.44		1.50		1.75		1.95		0.45		1.66		1.68		1.61		1.60	

TM-LVI was an independent prognostic factor for relapse-free survival of colon cancer in stage IIa, IIIb, and IIIc.

M/F, Male/Female; Diff/Undiff, Differentiated/Undifferentiated; LN, Lymph node; RR, Risk ratio; Bold type, *P *< 0.05.

**Table 3:** Univariate and multivariate analysis for relapse-free survival of rectal cancer was performed in each clinical stage.

Stage	I				IIa				IIb				IIc		IIIa				IIIb				IIIc	
Factors	Univariate analysis		Multivariate analysis		Univariate analysis		Multivariate analysis		Univariate analysis		Multivariate analysis		Univariate analysis		Univariate analysis		Multivariate analysis		Univariate analysis		Multivariate analysis		Univariate analysis	
	RR	*P*	RR	*P*	RR	*P*	RR	*P*	RR	*P*	RR	*P*	RR	*P*	RR	*P*	RR	*P*	RR	*P*	RR	*P*	RR	*P*
Age																								
75≥/<75	1.66	**0.048**	1.52	0.10	1.16	0.43	—	—	0.92	0.83			0.86	0.77	2.36	**0.028**	1.74	0.16	1.09	0.55	—	—	0.95	0.85
Gender																								
M/F	0.84	0.38	—	—	1.27	0.12	—	—	0.90	0.71			1.37	0.35	1.79	0.07			1.27	**0.03**	1.24	0.054	1.15	0.39
Histology																								
Diff/Undiff	1.04	0.96	—	—	0.97	0.94	—	—	0.51	0.19	0.45	0.14	0.43	0.12	0.31	0.056			1.06	0.83	—	—	0.64	**0.049**
Dissected LN																								
12 > /12≤	1.38	0.13	—	—	1.11	0.55	—	—	1.54	0.2.			1.86	0.24	0.98	0.95			1.60	**0.0007**	1.54	**0.007**	0.85	0.90
Adjuvant																								
Yes/No	1.99	0.089	—	—	1.46	**0.029**	1.36	0.07	1.27	0.44			1.00	1.00	0.42	**0.006**	0.53	0.053	0.75	**0.014**	0.76	**0.02**	0.90	0.56
TM-LVI																								
C/A	3.40	**<0.0001**	3.24	**0.0002**	2.66	**<0.0001**	2.59	**<0.0001**	2.98	**0.0004**	3.25	**0.0003**	1.22	0.50	5.61	**0.0005**	4.15	**0.002**	2.13	**<0.0001**	2.07	**<0.0001**	1.30	0.06
B/A	2.29		2.24		1.80		1.77		0.88		0.98		0.74		2.33		2.42		1.37		1.32		0.84	
C/B	1.49		1.45		1.48		1.47		3.38		3.30		1.65		2.41		1.71		1.56		1.56		1.54	

TM-LVI was an independent prognostic factor for relapse-free survival of rectal cancer in stage I, IIa, IIb, IIIa, and IIIb.

M/F, Male/Female; Diff/Undiff, Differentiated/Undifferentiated; LN, Lymph node; RR, Risk ratio; Bold type, *P *< 0.05.

**Table 4:** Univariate and multivariate analysis for disease specific survival of colon cancer was performed in each clinical stage.

Stage	I		IIa				IIb				IIc		IIIa		IIIb				IIIc			
Factors	Univariate analysis		Univariate analysis		Multivariate analysis		Univariate analysis		Multivariate analysis		Univariate analysis		Univariate analysis		Univariate analysis		Multivariate analysis		Univariate analysis		Multivariate analysis	
	RR	*P*	RR	*P*	RR	*P*	*RR*	*P*	RR	*P*	RR	*P*	RR	*P*	RR	*P*	RR	*P*	RR	*P*	RR	*P*
Age																						
75≥/<75	1.15	0.83	2.21	**0.0006**	2.01	**0.0025**	1.97	0.051	1.78	0.10	1.79	0.2	1.49	0.64	1.35	0.052			1.30	0.20		
Gender																						
M/F	1.58	0.44	1.39	0.14			0.80	0.49			1.00	1.0	0.88	0.85	1.42	**0.01**	1.41	**0.02**	1.10	0.57		
Histology																						
Diff/Undiff	6.8×10^8^	0.44	5.23	**0.026**	4.67	**0.04**	2.00	0.29			1.45	0.6	1.9×10^9^	0.37	0.75	0.26			0.61	**0.02**	0.63	**0.03**
Dissected LN																						
12 > /12≤	0.65	0.64	2.64	**0.0002**	2.21	**0.004**	1.41	0.64			1.26	0.68	ND	0.88	1.49	**0.049**	1.41	0.1	1.54	0.15		
Adjuvant																						
Yes/No	1.5×10^−8^	0.43	0.62	0.19			1.47	0.28			0.33	0.09	0.86	0.84	0.90	0.46			0.92	0.64		
TM-LVI																						
C/A	1.6×10^−9^	0.13	2.85	**0.0003**	2.80	**0.0009**	3.68	**0.003**	3.47	**0.006**	2.29	0.3	4.61	0.14	2.64	**<0.0001**	2.64	**<0.0001**	2.65	**<0.0001**	2.63	**<0.0001**
B/A	1.6×10^−9^		2.25		2.04		1.53		1.51		1.38		4.05		1.68		1.67		1.53		1.53	
C/B	1.0		1.27		1.37		2.41		2.30		1.66		1.14		1.57		1.58		1.73		1.72	

TM-LVI was an independent prognostic factor for relapse-free survival of colon cancer in stage IIa, IIb, IIIb, and IIIc.

M/F, Male/Female; Diff/Undiff, Differentiated/Undifferentiated; LN, Lymph node; RR, Risk ratio; Bold type, *P *< 0.05.

**Table 5:** Univariate and multivariate analysis for disease specific survival of rectal cancer was performed in each clinical stage.

Stage	I				IIa				IIb				IIc		IIIa				IIIb				IIIc			
Factors	Univariate analysis		Multivariate analysis		Univariate analysis		Multivariate analysis		Univariate analysis		Multivariate analysis		Univariate analysis		Univariate analysis		Multivariate analysis		Univariate analysis		Multivariate analysis		Univariate analysis		Multivariate analysis	
	RR	*P*	RR	*P*	RR	*P*	RR	*P*	RR	*P*	RR	*P*	RR	*P*	RR	*P*	RR	*P*	RR	*P*	RR	*P*	RR	*P*	RR	*P*
Age																										
75 ≥ /<75	3.02	**0.007**	2.68	**0.02**	2.52	**0.0007**	2.55	**0.0006**	1.14	0.81			0.78	0.73	1.90	0.28			1.69	**0.01**	1.30	0.23	1.59	0.13	1.28	0.43
Gender																										
M/F	1.61	0.19			1.50	0.10			0.56	0.17			1.74	0.23	3.17	**0.02**	2.57	0.07	1.09	0.58			1.19	0.39		
Histology																										
Diff/Undiff	1.8×10^9^	0.21			0.88	0.86			0.62	0.55			0.32	0.07	0.18	**0.03**	0.22	**0.047**	1.16	0.67			0.67	0.17	0.69	0.22
Dissected LN																										
12 > /12≤	2.27	**0.04**	2.05	0.07	1.49	0.15			2.86	**0.044**	2.73	0.06	3.07	0.08	1.15	0.86			1.83	**0.006**	1.67	**0.03**	1.24	0.54		
Adjuvant																										
Yes/No	1.21	0.80			1.48	0.16			1.36	0.49			2.02	0.12	0.39	**0.04**	0.45	0.1	0.63	**0.005**	0.71	0.06	0.68	0.1	0.70	0.13
TM-LVI																										
C/A	2.34	**0.004**	1.94	**0.01**	3.69	**<0.0001**	3.75	**<0.0001**	4.55	**0.0003**	4.32	**0.0004**	2.20	0.35	7.25	**0.007**	5.10	**0.037**	3.26	**<0.0001**	3.16	**<0.0001**	1.52	**0.01**	1.52	**0.01**
B/A	3.40		3.12		1.94		1.92		0.69		0.67		1.49		2.39		2.00		1.75		1.72		0.73		0.74	
C/B	0.69		0.62		1.90		1.95		6.57		6.46		1.47		3.04		2.54		1.86		1.84		2.07		2.06	

TM-LVI was an independent prognostic factor for relapse-free survival of rectal cancer in stage I, IIa, IIb, IIIa, IIIb, and IIIc.

M/F, Male/Female; Diff/Undiff, Differentiated/Undifferentiated; LN, Lymph node; RR, Risk ratio; Bold type, *P *< 0.05.

### Association between the adjuvant therapy and TM-LVI status

The application of adjuvant therapy significantly differed by TM-LVI in stage I, IIa, IIIa, and IIIc CC and stage IIIa RC (Table 6). However, the application of adjuvant therapy was not irrelevant with the recurrence risk evaluated by TM-LVI except for stage IIa CC. The application of adjuvant therapy did not differ by TM-LVI status (stage IIIb CC and in stage I, IIa, IIb, and IIIb RC) or adversely decreased in spite of the increased recurrence (stage IIIc CC and stage IIIa RC), although TM-LVI status was an independent prognostic factor for both RFS and DSS in these stages. These results suggested that TM-LVI, which represents tumour marker elevation and lymphovascular invasion, was not used for determining the use of adjuvant treatment.

**Table 6:** Association between the adjuvant therapy and TM-LVI status was assessed in each clinical stage.

Location	TNM stage	TM-LVI			*P*
		A Number (%)	B Number (%)	C Number (%)	
Colon	I	35(2.1%)	5(2.0%)	4(8.5%)	**0.013**
	IIa	148(11.3%)	85(14.1%)	44(18.5%)	**0.0054**
	IIb	44(23.4%)	33(21.3%)	17(19.8%)	0.77
	IIc	15(19.5%)	14(21.2%)	11(22.9%)	0.90
	IIIa	145(67.1%)	46(62.2%)	5(31.3%)	**0.014**
	IIIb	398(61.1%)	401(66.7%)	261(62.3%)	0.10
	IIIc	58(80.6%)	112(70.9%)	130(63.4%)	**0.0003**
Rectum	I	45(3.9%)	14(6.3%)	2(4.0%)	0.25
	IIa	86(14.8%)	85(19.4%)	37(21.1%)	0.059
	IIb	12(20.7%)	26(34.2%)	11(22.5%)	0.16
	IIc	9(26.5%)	8(26.7%)	13(44.8%)	0.22
	IIIa	151(77.8%)	72(73.5%)	11(52.4%)	**0.036**
	IIIb	272(73.5%)	293(72.7%)	225(68.8%)	0.34
	IIIc	34(66.7%)	68(75.6%)	117(75.0%)	0.45

The application of adjuvant therapy was not related to the risk of recurrence as estimated by TM-LVI status except for stage IIa CC. Red circles indicated that TM-LVI was an independent prognostic factor for both relapse-free survival and disease specific survival. Adjuvant therapy may be recommended according to TM-LVI status in these stages.

Bold type, *P *< 0.05.

## Discussion

Due to the heterogeneity of the disease, further classification beyond TNM-based clinical staging has been considered indispensable for determining the treatment strategy of CRC. Despite continued effort, novel modalities are still under development^10–13^. We combined TM elevation and LVI for subtyping of the TNM staging system because of their potential as prognostic factors and ready-to-use availability. Among candidate classifications, we selected the most statistically suitable classification and named TM-LVI.

Our data demonstrated that TM-LVI was useful for subtyping and prognosis for not only RFS but also DSS, although we picked up TM-LVI depending on RFS. This may be consistent with the fact that TM elevation and LVI have been considered prognostic factors for RFS and overall survival, respectively^16–18,21,22^.

Our data indicated that adjuvant treatment was not considered in accordance with the recurrence risk determined by TM-LVI status. Thus, TM-LVI may be useful for considering adjuvant therapy after curative surgery when TM-LVI is an independent prognostic factor for both RFS and DSS (stage IIa, IIIb, and IIIc CC and stage I, IIa, IIb, IIIa, and IIIb RC).

We evaluated LVI by scoring both lymphatic invasion and venous invasion, although LVI is usually discussed as positive or negative. This may be because pathological assessments differ among pathologists regarding LVI status. In our massive dataset, the influence of pathologists may be reduced compared to data from single institute.

Our study has several limitations. First, the pathological results of LVI were not discussed among the pathologists to standardize the evaluation of LVI. Second, in this retrospective study, the treatment of the patients may vary depending on the clinicians and the hospitals. Third, genetic information was not collected. MSI status, which is associated with the prognosis of CRC patients, is not routinely assessed in most Japanese hospitals^25^.

In conclusion, we present a cost and labour efficient subtyping method (TM-LVI) for CRC patients using clinicopathological features routinely assessed in the clinic all over the world. The usefulness of TM-LVI should be validated in the future by randomized clinical trials regarding adjuvant treatment after curative surgery for patients with poor prognosis as estimated by TM-LVI.

## Methods

### Patients and data collection

The JFUP-CRC contains data from twenty-three institutes in Japan (Sapporo Medical University Hospital, Hirosaki University Hospital, Niigata University Hospital, Niigata Cancer Center Hospital, National Defence Medical College Hospital, Tochigi Cancer Center Hospital, Tokyo University Hospital, Tokyo Metropolitan Cancer and Infectious Diseases Center Komagome Hospital, National Cancer Center Hospital, Tokyo Women’s Medical University Hospital, National Center for Global Health and Medicine Hospital, Tokyo Medical and Dental University Hospital, Keio University Hospital, Teikyo University Hospital, Kyorin University Hospital, Kitasato University Hospital, Fujita Health University Hospital, Aichi Cancer Center Hospital, Kyoto University Hospital, Osaka International Cancer Institute Hospital, Osaka Rosai Hospital, Hyogo College of Medicine Hospital, and Kurume University Hospital). Each hospital retrospectively collected the clinical data of patients with CRC who underwent curative surgery. This study was approved by the institutional review board or ethics committee at all 23 hospitals above and was conducted in accordance with the Declaration of Helsinki and Ethical Guidelines for Clinical Research. The patients provided written informed consent, and patients had the option to opt-out if there was any disagreement with this study. The JFUP-CRC office pooled and organized the data for this study. Among the patients whose data are contained in the database, we assessed 11771 patients, consisting of 7151 CC patients and 4620 RC patients, who received curative surgery between 2004 and 2008. We classified these patients by the 8th edition TNM staging system^6,7^. A higher level of CEA or CA19-9 than the upper limit in each hospital was determined to indicate TM elevation. TM elevation was classified into three categories: both CEA and CA19-9 elevation, either CEA or CA19-9 elevation, or no elevation. Lymphatic or venous invasion was evaluated as 0 (no invasion), 1 (minimal invasion), 2 (moderate invasion), or 3 (severe invasion) by pathologists in each hospital according to the classification by the JSCCR^8^. We summed the evaluation in both lymphatic invasion and venous invasion as LVI, which was categorized as none (0), slight (1-2), mild (3-4), or severe (5-6).

### Selection of the most suitable subtyping

To select the most suitable subtyping using both TM elevation and LVI, we assessed six candidate classifications (ABC1-ABC5, AB), which simplified 12 categories determined by TM elevation (both, either, or none) and LVI (none, slight, mild, or severe) into three (A, B, and C; ABC1-ABC5) or two (A, B; AB) subtypes (Table 1). Then, Akaike’s information criterion (AIC) and Harrell’s concordance index (c-index) were derived from the Cox proportional hazard model with TNM and each subtype to explore the most suitable (lower AIC and/or higher c-index) subtyping for RFS. If the interaction term between TNM and the candidate classification was significant, the term was included in the Cox model for ranking the incidence rates of RFS within each subtype. We did not exclude the patients who received adjuvant therapy, because we explored the subtyping available in all patients who received curative surgery.

### Validation of TM-LVI for RFS and DSS

Univariate analysis was performed using Cox proportional hazard model, along with age (75 ≤ or not), sex, histological type (differentiated type or not), number of dissected lymph nodes (12 ≤ or not), and adjuvant therapy. Multivariate analysis was also performed using Cox proportional hazard model with the factors that showed significant differences (p < 0.05) in the univariate analysis. When TM-LVI was the only significant factor in the univariate analysis, multivariate analysis was performed using Cox proportional hazard model using the factors with p < 0.2. The influence of TM-LVI on RFS and DSS in each TNM stage was also assessed by Kaplan-Meier curve and evaluated by the Log-rank test.

### Data analysis

The comparisons of the clinicopathological characteristics between CC and RC patients were assessed by the chi-squared test or t-test. The influence of the clinicopathological features on RFS and DSS was evaluated by a Cox proportional hazard model. RFS and DSS was calculated by the Kaplan-Meier method and compared by the log-rank test. Differences in the application of adjuvant therapy by combined subtyping were evaluated by the chi-squared test. Multivariate analysis for RFS and DSS was performed using a Cox proportional hazard model. A *P* value of <0.05 was considered significant for all analyses. All statistical analyses were performed using SAS software (SAS Institute, Cary, NC, USA).

## Supplementary information

Supplementary information.
