# Supplementary material for: Combination of preoperative tumour markers and lymphovascular invasion with TNM staging as a cost and labour efficient subtyping of colorectal cancer
Source: Sci Rep. 2020 Jun 24;10:10238. doi: 10.1038/s41598-020-66652-z (PMC7314851; doi:10.1038/s41598-020-66652-z)
Supplement: Supplementary file 1 — Supplementary information. [file 41598_2020_66652_MOESM1_ESM.docx]

Submission ID 8e262be1-0e11-4a88-b859-ba2e5dd179cd

Combination of preoperative tumour markers and lymphovascular invasion with TNM staging as a cost and labour efficient subtyping of colorectal cancer.

Tomoki Yamano, Shinichi Yamauchi, Masataka Igeta, Yuya Takenaka, Jihyung Song, Kei Kimura, Michiko Yasuhara, Akihito Babaya, Kozo Kataoka, Naohito Beppu, Masataka Ikeda, Naohiro Tomita, Kenichi Sugihara

Supplementary Tables

| Clinicopathological features | Colon cancer  (N=7151) | Rectal cancer  (N=4620) | *P* |
| --- | --- | --- | --- |
| Age: mean (SD) | 67.3 (11.12) | 63.1 (11.32) | **<0.0001** |
| Gender: N (%) |  |  | **<0.0001** |
| Male | 4047 (56.6) | 2904 (62.9) |  |
| Female | 3104 (43.4) | 1716 (37.1) |  |
| Histology: N (%) |  |  | **<0.0001** |
| Differentiated | 6718 (93.9) | 4441 (96.1) |  |
| Poorly | 222 (3.1) | 79 (1.7) |  |
| Mucinous | 211 (3.0) | 100 (2.2) |  |
| Lymphatic invasion: N (%) |  |  | **0.0027** |
| ly0 | 3206 (44.8) | 1920 (41.6) |  |
| ly1 | 2892 (40.4) | 1998 (43.3) |  |
| ly2 | 896 (12.5) | 612 (13.3) |  |
| ly3 | 157 (2.2) | 90 (2.0) |  |
| Venous invasion: N (%) |  |  | **<0.0001** |
| v0 | 2918 (40.8) | 1598 (34.6) |  |
| v1 | 2689 (37.6) | 1699 (36.8) |  |
| v2 | 1225 (17.1) | 1008 (21.8) |  |
| v3 | 319 (4.5) | 315 (6.8) |  |
| Lymphovascular invasion: N (%) |  |  | **<0.0001** |
| none | 1821 (25.5) | 927 (20.1) |  |
| slight | 3662 (51.2) | 2381 (51.5) |  |
| mild | 1520 (21.3) | 1172 (25.4) |  |
| severe | 148 (2.1) | 140 (3.0) |  |
|  |  |  |  |
| Dissected LN number: N (%) |  |  | **<0.0001** |
| 12≤ | 5383 (75.3) | 3562 (77.1) |  |
| <12 | 1700 (23.8) | 976 (21.1) |  |
| Unknown | 68 (0.9) | 82 (1.8) |  |
|  |  |  |  |
| TNM stage: N (%) |  |  | **<0.0001** |
| I | 1965 (27.5) | 1438 (31.1) |  |
| IIa | 2154 (30.1) | 1196 (25.9) |  |
| IIb | 429 (6.0) | 183 (4.0) |  |
| IIc | 191 (2.7) | 93 (2.0) |  |
| IIIa | 306 (4.3) | 313 (6.8) |  |
| IIIb | 1671 (23.4) | 1100 (23.8) |  |
| IIIc | 435 (6.1) | 297 (6.4) |  |
| CEA elevation: N (%) | 2048 (28.6%) | 1428 (30.9%) | **0.0084** |
| CA19-9 elevation: N (%) | 908 (12.7%) | 568 (12.3%) | 0.52 |
| Tumour marker elevation: N (%) |  |  | **0.025** |
| both | 553 (7.7) | 348 (7.5) |  |
| either | 1850 (25.9) | 1300 (28.1) |  |
| none | 4748 (66.4) | 2972 (64.3) |  |
| Adjuvant therapy: Yes; N (%) | 2011 (28,1) | 1591 (34.4) | **<0.0001** |
| 5-fluruorouracil based chemotherapy | 1915 (95.2) | 1494 (93.9) | **0.004** |
| Oxaliplatin based chemotherapy | 29 (1.4) | 30 (1.9) |  |
| Irinotecan based chemotherapy | 16 (0.8) | 24 (1.5) |  |
| Chemoradiation | 51 (2.5) | 34 (2.1) |  |
| Radiation | 0 | 7 (0.4) |  |
| Unknown | 0 | 2 (0.1) |  |
| Adjuvant therapy by TNM stage: Yes  N (%) |  |  |  |
| I | 44 (2.2) | 61 (4.2) | **0.0008** |
| IIa | 277 (12.9) | 208 (17.4) | **0.0004** |
| IIb | 94 (21.9) | 49 (26.8) | 0.2 |
| IIc | 40 (20.9) | 30 (32.3) | **0.04** |
| IIIa | 196 (64.1) | 234 (74.8) | **0.0038** |
| IIIb | 1060 (63.4) | 790 (71.8) | **<0.0001** |
| IIIc | 300 (69.0) | 219 (73.7) | 0.16 |

**Supplementary Table 1.** Clinicopathological features by tumour location. There were significant differences between colon cancer and rectal cancer in clinicopathological features. Then, we assessed the data by colon cancer and rectal cancer. CA19-9, cancer antigen 19-9; CEA, carcinoembryonic antigen; LN, lymph node; N, number; Bold type, P<0.05.

| **Location** | **Candidate** | **Model** ^a^ | **AIC** |
| --- | --- | --- | --- |
| Colon | ABC1 | Multivariate main effect (TNM, ABC) | 16573.570 |
|  | ABC2 | Multivariate main effect (TNM, ABC) | 16574.606 |
|  | ABC4 | Multivariate main effect (TNM, ABC) | 16575.448 |
|  | ABC1 | Multivariate interaction (TNM, ABC, TNMxABC) | 16583.052 |
|  | ABC2 | Multivariate interaction (TNM, ABC, TNMxABC) | 16584.259 |
|  | ABC4 | Multivariate interaction (TNM, ABC, TNMxABC) | 16585.769 |
|  | ABC5 | Multivariate main effect (TNM, ABC) | 16590.450 |
|  | ABC3 | Multivariate main effect (TNM, ABC) | 16591.953 |
|  | ABC3 | Multivariate interaction (TNM, ABC, TNMxABC) | 16597.007 |
|  | ABC5 | Multivariate interaction (TNM, ABC, TNMxABC) | 16597.966 |
|  | AB | Multivariate main effect (TNM, ABC) | 16602.067 |
|  | AB | Multivariate interaction (TNM, ABC, TNMxABC) | 16609.947 |
|  | ABC1 | Univariate (TNM) | 16674.719 |
|  | ABC2 | Univariate (TNM) | 16674.719 |
|  | ABC3 | Univariate (TNM) | 16674.719 |
|  | AB | Univariate (TNM) | 16674.719 |
|  | ABC4 | Univariate (TNM) | 16674.719 |
|  | ABC5 | Univariate (TNM) | 16674.719 |
|  | ABC2 | Univariate (ABC) | 17102.296 |
|  | ABC4 | Univariate (ABC) | 17104.662 |
|  | ABC1 | Univariate (ABC) | 17104.981 |
|  | ABC5 | Univariate (ABC) | 17154.139 |
|  | ABC3 | Univariate (ABC) | 17162.091 |
|  | AB | Univariate (ABC) | 17270.740 |
| Rectum | ABC1 | Multivariate interaction (TNM, ABC, TNMxABC) | 15167.344 |
|  | ABC1 | Multivariate main effect (TNM, ABC) | 15168.839 |
|  | ABC2 | Multivariate main effect (TNM, ABC) | 15169.616 |
|  | ABC2 | Multivariate interaction (TNM, ABC, TNMxABC) | 15170.495 |
|  | ABC4 | Multivariate main effect (TNM, ABC) | 15174.613 |
|  | ABC4 | Multivariate interaction (TNM, ABC, TNMxABC) | 15176.075 |
|  | ABC5 | Multivariate main effect (TNM, ABC) | 15177.877 |
|  | ABC3 | Multivariate main effect (TNM, ABC) | 15182.590 |
|  | ABC5 | Multivariate interaction (TNM, ABC, TNMxABC) | 15191.000 |
|  | ABC3 | Multivariate interaction (TNM, ABC, TNMxABC) | 15193.736 |
|  | AB | Multivariate main effect (TNM, ABC) | 15195.631 |
|  | AB | Multivariate interaction (TNM, ABC, TNMxABC) | 15198.272 |
|  | ABC1 | Univariate (TNM) | 15257.394 |
|  | ABC2 | Univariate (TNM) | 15257.394 |
|  | ABC3 | Univariate (TNM) | 15257.394 |
|  | AB | Univariate (TNM) | 15257.394 |
|  | ABC4 | Univariate (TNM) | 15257.394 |
|  | ABC5 | Univariate (TNM) | 15257.394 |
|  | ABC2 | Univariate (ABC) | 15403.906 |
|  | ABC1 | Univariate (ABC) | 15404.052 |
|  | ABC4 | Univariate (ABC) | 15414.195 |
|  | ABC5 | Univariate (ABC) | 15436.808 |
|  | ABC3 | Univariate (ABC) | 15448.626 |
|  | AB | Univariate (ABC) | 15509.363 |

**Supplementary Table 2.** Akaike’s information criterion (AIC) values by candidate subtyping.

^a^ AIC was calculated using the Cox models listed above. Multivariate interaction models included the interaction term between TNM and ABC, TNMxABC, in addition to the main effects of TNM and ABC. Multivariate main effect models included TNM and ABC as main effects. Univariate models included TNM or ABC as a main effect. ABC1 showed the lowest AIC according to the models including TNM staging.

| **Location** | **Candidate** | **Model** ^a^ | **C-index** |
| --- | --- | --- | --- |
| Colon | ABC1 | Multivariate interaction (TNM, ABC, TNMxABC) | 0.7756 |
|  | ABC1 | Multivariate main effect (TNM, ABC) | 0.7745 |
|  | ABC2 | Multivariate interaction (TNM, ABC, TNMxABC) | 0.7743 |
|  | ABC4 | Multivariate interaction (TNM, ABC, TNMxABC) | 0.7739 |
|  | ABC2 | Multivariate main effect (TNM, ABC) | 0.7736 |
|  | ABC4 | Multivariate main effect (TNM, ABC) | 0.7733 |
|  | ABC3 | Multivariate interaction (TNM, ABC, TNMxABC) | 0.7691 |
|  | ABC5 | Multivariate interaction (TNM, ABC, TNMxABC) | 0.7691 |
|  | ABC5 | Multivariate main effect (TNM, ABC) | 0.7685 |
|  | ABC3 | Multivariate main effect (TNM, ABC) | 0.7683 |
|  | AB | Multivariate interaction (TNM, ABC, TNMxABC) | 0.7649 |
|  | AB | Multivariate main effect (TNM, ABC) | 0.7647 |
|  | ABC1 | Univariate (TNM) | 0.7541 |
|  | ABC2 | Univariate (TNM) | 0.7541 |
|  | ABC3 | Univariate (TNM) | 0.7541 |
|  | AB | Univariate (TNM) | 0.7541 |
|  | ABC4 | Univariate (TNM) | 0.7541 |
|  | ABC5 | Univariate (TNM) | 0.7541 |
|  | ABC1 | Univariate (ABC) | 0.6789 |
|  | ABC2 | Univariate (ABC) | 0.6774 |
|  | ABC4 | Univariate (ABC) | 0.6766 |
|  | ABC3 | Univariate (ABC) | 0.6532 |
|  | ABC5 | Univariate (ABC) | 0.6481 |
|  | AB | Univariate (ABC) | 0.5987 |
| Rectum | ABC1 | Multivariate interaction (TNM, ABC, TNMxABC) | 0.7265 |
|  | ABC2 | Multivariate interaction (TNM, ABC, TNMxABC) | 0.7250 |
|  | ABC4 | Multivariate interaction (TNM, ABC, TNMxABC) | 0.7238 |
|  | ABC1 | Multivariate main effect (TNM, ABC) | 0.7236 |
|  | ABC2 | Multivariate main effect (TNM, ABC) | 0.7232 |
|  | ABC4 | Multivariate main effect (TNM, ABC) | 0.7222 |
|  | ABC5 | Multivariate interaction (TNM, ABC, TNMxABC) | 0.7210 |
|  | ABC3 | Multivariate interaction (TNM, ABC, TNMxABC) | 0.7210 |
|  | ABC5 | Multivariate main effect (TNM, ABC) | 0.7187 |
|  | ABC3 | Multivariate main effect (TNM, ABC) | 0.7185 |
|  | AB | Multivariate interaction (TNM, ABC, TNMxABC) | 0.7158 |
|  | AB | Multivariate main effect (TNM, ABC) | 0.7142 |
|  | ABC1 | Univariate (TNM) | 0.7000 |
|  | ABC2 | Univariate (TNM) | 0.7000 |
|  | ABC3 | Univariate (TNM) | 0.7000 |
|  | AB | Univariate (TNM) | 0.7000 |
|  | ABC4 | Univariate (TNM) | 0.7000 |
|  | ABC5 | Univariate (TNM) | 0.7000 |
|  | ABC1 | Univariate (ABC) | 0.6594 |
|  | ABC2 | Univariate (ABC) | 0.6582 |
|  | ABC4 | Univariate (ABC) | 0.6548 |
|  | ABC3 | Univariate (ABC) | 0.6343 |
|  | ABC5 | Univariate (ABC) | 0.6315 |
|  | AB | Univariate (ABC) | 0.5948 |

**Supplementary Table 3.** Harrell’s concordance index (c-index) by candidate subtyping.

^a^ C-index was calculated using the Cox models listed above. Multivariate interaction models included the interaction term between TNM and ABC, TNMxABC, in addition to the main effects of TNM and ABC. Multivariate main effect models included TNM and ABC as main effects. Univariate models included TNM or ABC as a main effect.

ABC1 showed the highest C-index according to the models combined including TNM staging.

| Location | TNM staging | TM-LVI | | |
| --- | --- | --- | --- | --- |
|  |  | A | B | C |
| Colon cancer | I | 1 | 2 | 3 |
|  | IIa | 5 | 7 | 10 |
|  | IIb | 8 | 13 | 16 |
|  | IIc | 12 | 15 | 19 |
|  | IIIa | 4 | 6 | 9 |
|  | IIIb | 11 | 14 | 18 |
|  | IIIc | 17 | 20 | 21 |
| Rectal cancer | I | 1 | 4 | 5 |
|  | IIa | 3 | 8 | 11 |
|  | IIb | 9 | 7 | 19 |
|  | IIc | 15 | 12 | 18 |
|  | IIIa | 2 | 6 | 14 |
|  | IIIb | 10 | 13 | 16 |
|  | IIIc | 20 | 17 | 21 |

**Supplementary Table 4.** Ranking of relapse-free survival (RFS) by TM-LVI and TNM staging. Category C by TM-LVI belonged to the highest recurrence risk group in each TNM staging. Red circle indicated ranking 1 to 10 of good RFS. RFS was not ordered by TNM staging.
